# Cortical Surface Reconstruction from High-Resolution MR Brain Images

**DOI:** 10.1155/2012/870196

**Published:** 2012-02-01

**Authors:** Sergey Osechinskiy, Frithjof Kruggel

**Affiliations:** Department of Biomedical Engineering, University of California, Irvine, CA 92697, USA

## Abstract

Reconstruction of the cerebral cortex from magnetic resonance (MR) images is an important step in quantitative analysis of the human brain structure, for example, in sulcal morphometry and in studies of cortical thickness. Existing cortical reconstruction approaches are typically optimized for standard resolution (~1 mm) data and are not directly applicable to higher resolution images. A new PDE-based method is presented for the automated cortical reconstruction that is computationally efficient and scales well with grid resolution, and thus is particularly suitable for high-resolution MR images with submillimeter voxel size. The method uses a mathematical model of a field in an inhomogeneous dielectric. This field mapping, similarly to a Laplacian mapping, has nice laminar properties in the cortical layer, and helps to identify the unresolved boundaries between cortical banks in narrow sulci. The pial cortical surface is reconstructed by advection along the field gradient as a geometric deformable model constrained by topology-preserving level set approach. The method's performance is illustrated on exvivo images with 0.25–0.35 mm isotropic voxels. The method is further evaluated by cross-comparison with results of the FreeSurfer software on standard resolution data sets from the OASIS database featuring pairs of repeated scans for 20 healthy young subjects.

## 1. Introduction

Cortical reconstruction, the derivation of a computerized representation of the cerebral cortical layer based on three-dimensional (3D) magnetic resonance (MR) images of the brain, is an important step in quantitative analysis of the human brain structure, for example, in the analysis of cortical folding patterns, in brain morphometry, and in cortical thickness studies. Cortical surface models typically serve as a reference basis for all further analysis and therefore must be geometrically accurate and topologically correct in order to provide valid and accurate quantitative measures of brain structure [1].

**Figure 1:**
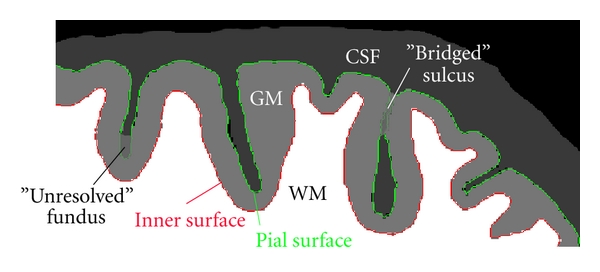
Schematic illustration of a fragment of brain slice. Contours of the inner and pial surface are marked in red and green. Due to partial volume effects and limited resolution, adjacent banks of gray matter in some sulci may appear as fused together, creating either a “bridged” sulcus or an unresolved sulcal fundus (a “buried” sulcus). Note that a “bridged” sulcus creates a topological defect, a handle, which may be corrected by a topology-preserving model, whereas a “buried” sulcus does not change the topology.

The cerebral cortex, considered at the spatial scale of MR images, is a thin layer of neural tissue, called gray matter (GM), located on the outer side of the white matter (WM), and surrounded by the cerebrospinal fluid (CSF). The cortex has a complex geometry of a highly folded layer with spatially varying curvature and thickness (thickness range 1–5 mm, average *≈*2.5 mm, see [1]). The cortical layer on a brain hemisphere can be represented as the inner space between two cortical surfaces (i.e., an inner surface at the WM/GM and an outer or pial surface at the GM/CSF interface, see Figure 1). It is a useful simplification to consider each surface as topologically equivalent to a 3D sphere. In practice, limited spatial resolution of MR images, noise, intensity inhomogeneities, and partial volume effects can all be the sources of geometrical inaccuracies and topological errors in the reconstructed cortical model. In particular, the opposite banks of gray matter in deep sulci are not always resolved as separate and can appear as fused together (Figure 1), leading to invalid models of the cortical layer and propagating errors further into quantitative measurements (e.g., cortical thickness). This may present a particular challenge for an automated reconstruction algorithm, requiring specific means for an automatic detection and correction of topologically and geometrically problematic cases.

Reconstruction of cortical surface models received considerable attention in neuroimaging research. Here, we only briefly overview some state-of-the-art methods; please refer to Han et al. [1] and Kim et al. [2] for additional discussion. A suite of algorithms for automated cortical reconstruction is implemented in the popular and freely available FreeSurfer software [3, 4]. FreeSurfer includes an algorithm for finding and correcting the topological defects in the initial WM/GM surface [5] and a method to deform the mesh for reconstructing the inner and pial surfaces. The deformable model is constrained by a second-order smoothing term [6] and by a mesh self-intersection prevention routine [3], which both help to resolve the boundaries between adjacent banks in tight sulci. The FreeSurfer automated toolchain is optimized for standard resolution T1-weighted MR images and conforms input data to 1 mm isotropic voxel size, as a rule. This is consistent with the fact that mesh self-intersection detection and prevention is computationally expensive (see [1, 6]) and does not scale well with increasing mesh resolution. Xu et al. [7] developed a deformable mesh model for reconstruction of the central cortical surface. The model deforms the topology-corrected initial WM/GM interface by forces derived from a smoothed gradient field [8] that was computed from a GM class membership function. The model does not perform a time-consuming check of mesh self-intersections, which is arguably less critical for finding the central surface, compared to the pial surface. Kim et al. [2] presented a different deformable mesh-based approach for reconstruction of a pial surface, which is called constrained Laplacian anatomic segmentation using proximity, or CLASP. The algorithm computes a Laplacian field mapping between the GM/WM interface and the skeleton of the partial volume classification of the CSF. The Laplacian map is then integrated into the deformable model's objective function, driving mesh vertices into locations with higher values of the Laplacian field. Terms for stretch and self-proximity are included to regularize the deforming mesh and prevent from mesh self-intersection inside sulci. The method by Kim et al. depends on accurate extraction of the CSF skeleton and therefore relies on an elaborate partial volume tissue classification algorithm. However, the accuracy of the Laplacian mapping may be compromised at locations, where the fused GM sulcal banks are not resolved. In addition, the computational cost of the self-proximity term may become prohibitive for high-resolution meshes. Zeng et al. [9] used implicit surfaces in a level set framework for simultaneous reconstruction of the inner and outer cortical surfaces coupled by the minimal and maximal distance constraint. However, this approach did not gain widespread use, because it does not preserve the topology of the evolving surfaces and, in some areas, the distance coupling term may suppress the data attachment term, resulting in geometrical inaccuracies [10]. Han et al. [1] described a method for automated reconstruction of cortical surfaces, called CRUISE, which is built around a geometric deformable model using level sets. To help resolve the cortical banks in sulci, a thin digital separating barrier is constructed using the anatomically consistent enhancement algorithm ACE [1, 11], which finds a skeleton of the weighted distance function computed from the Eikonal equation with a speed function modulated by the CSF class membership. At the core of the CRUISE method is a topology-preserving geometric deformable surface model, TGDM [1, 11, 12], which models the evolution of a level set function under the influence of signed pressure forces computed from tissue class membership values and curvature forces defined by the surface geometry. The central surface of the cortex is reconstructed by a TGDM with GGVF advection forces similar to those in Xu et al. [7].

We present a method, henceforth, designated dielectric layer field mapping, or DELFMAP, for the automated reconstruction of the cortical compartment from MR images, which is based on several partial differential equation (PDE) modeling stages. Our method is inspired by the work of Han et al. and uses a similar level set framework, but introduces a different perspective, consolidating all algorithmic stages around the key mathematical model of a potential field in an inhomogeneous dielectric medium. Our method scales well with image resolution and has an advantage over other existing methods in reconstruction from high-resolution MR images with submillimeter voxel sizes, because (1) in contrast to deformable mesh models in FreeSurfer or CLASP, it avoids the computational cost of testing for mesh self-intersection and self-proximity; (2) similarly to CRUISE, it uses an efficient narrow-band algorithm for the level set evolution; (3) in contrast to CRUISE that requires solving a system of three second-order PDEs in GGVF, our method solves just one second-order PDE and does not need an intermediate step of reconstructing a central cortical surface.

Preliminary results of this work were presented in two conference publications [13, 14]. This report expands on the methodology and experimental results and adds a validation study that performs cross-comparison of our method's cortical reconstruction results with those obtained using FreeSurfer [3, 4] on standard resolution data for 20 healthy young subjects (test-retest repeated scans) from the OASIS database [15].

## 2. Methods

The DELFMAP method proceeds as follows. A potential field is computed using the mathematical model of an electric field in an inhomogeneous dielectric medium, where the segmented WM poses as a charged conductive object and the classified GM poses as an inhomogeneous dielectric layer with permittivity proportional to GM class probability values. This electrostatic model serves the purpose of concentrating the flux of the mapping flow in a layer of voxels classified as GM and helps to identify the separating barriers between cortical banks in sulci, where the mapping flow collides. Correspondence trajectories following the lines of the potential field and geodesic distances from WM boundary are determined using PDEs, and a digital skeleton of the sulcal medial surface separating GM sulcal banks is derived by finding collisions in the correspondence trajectories and shocks in the distance field. The computed electric field retains the desired laminar properties of the Laplacian mapping in the bulk of the cortical layer and is used as the potential flow that maps the inner surface to the outer. The outer (pial) cortical surface is reconstructed using a geometric deformable model level set framework [16] with an advection along the gradient of the potential field, which is constrained by the identified skeleton of the sulcal medial surfaces and (optionally) by a maximal distance/proximity constraint.

### 2.1. Image Processing Chain

**Figure 2:**
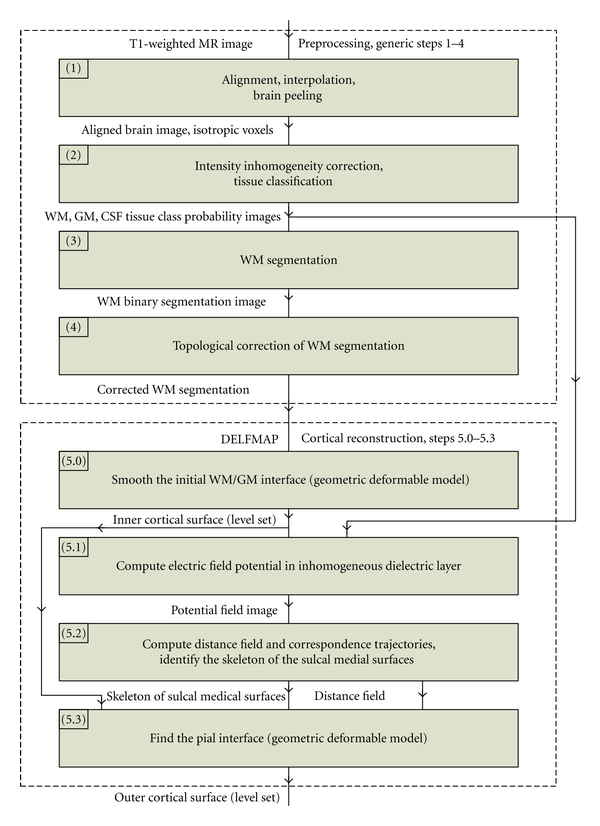
Block diagram of the overall image processing chain, where the DELFMAP method addresses the reconstruction of cortical surfaces (steps 5.0–5.3) after the preprocessing stage (steps 1–4).

DELFMAP takes as input a set of volumetric images containing WM and GM tissue class probability/membership functions and a refined WM model, supplied either as a topology-corrected WM binary segmentation or as a WM/GM interface level set function. The overall chain of general image processing steps is outlined as follows (Figure 2) (1) A T1-weighted volumetric MR image is (optionally) aligned with the stereotaxic coordinate system, interpolated to isotropic voxel size, and is preprocessed with a brain-peeling algorithm that derives a mask of voxels related to the cerebral tissues only. (2) The brain image is corrected for intensity inhomogeneities and is classified into WM,GM,CSF/background probability images. (3) A raw WM binary segmentation is derived from the class probability images (by thresholding or a maximum-probability rule), and brain stem and cerebellum are (optionally) removed from the WM segmentation. (4) A topology-corrected WM volume is obtained from the raw WM binary segmentation by an automated algorithm or by manual editing, or a combination of both. (5) DELFMAP uses the output of step 2 and step 4 to reconstruct the inner and outer cortical surfaces. We note that steps 1–4 are common to many brain MR image processing workflows, therefore DELFMAP can be easily integrated with a wide variety of toolchains. More specifically, we used processing steps described in Yang and Kruggel [17] in our experiments with 3-Tesla in-vivo images, and we applied algorithms described in Kruggel et al. [18] for the analysis of exvivo high-resolution images. In step 4, for exvivo images, we used manual editing for filling ventricles and correcting large topological defects, and we applied a topological region-growing algorithm similar to the one in Kriegeskorte and Goebel [19] to obtain a genus zero WM binary object. In cross-validation with FreeSurfer on the OASIS data sets, we used the FreeSurfer's processing toolchain for the initial steps that are common between the two methods (i.e., steps 1–4 that lead to a topologically-corrected WM segmentation); therefore, the cross-method comparison of cortical reconstructions is not confounded by differences in preprocessing approaches. Finally, we emphasize that, in all our experiments involving DELFMAP, the tissue classification was performed by a modified version (see [18]) of the adaptive fuzzy clustering algorithm [20] augmented with a spatial regularization term [1]; this also applies to GM and WM tissue classification that was used by DELFMAP in cross-validation study on the OASIS data sets.

### 2.2. Inner Cortical Surface

The inner cortical surface is reconstructed by a deformable model (Figure 2, step 5.0) that smooths the initial WM/GM interface, which is determined by the corrected WM segmentation. For this purpose, we use a topology-preserving geometric deformable model (similar to [12]), which is described in detail in Section 2.6. For smoothing, we typically run 2-3 iterations of the deformable model with the mean curvature term only. We will denote the “inside” region of the level set function representing the inner cortical surface by *Ω*_*w*_.

### 2.3. Electric Field Model

A potential field is found as a solution to the PDE modeling an electric field around a charged conductive object (WM) insulated by a dielectric layer (GM) having spatially inhomogeneous electric permittivity, which is set proportional to GM tissue class probability (Figure 2, step 5.1). In such a model, the flux of the electric field is confined in regions of higher permittivity, that is, where GM class probability is higher; therefore, trajectories following the lines of the electric field trace through the GM layer before exiting into the background space. Thus, the flux of the mapping flow is concentrated in a layer of voxels classified as GM. Let *Ω* denote the 3D image domain with the boundary Γ(*Ω*). We will denote WM and GM tissue class probability images by $P_{w}(\vec{r}\;)$ and $P_{g}(\vec{r}\;)$ ($\vec{r}\in \Omega$), where $\vec{r}=(x,y,z)$ is a 3D point. Let $\varphi (\vec{r}\;)$ denote a potential field, a scalar function defined over *Ω*. Let $ɛ(\vec{r}\;)$ denote another scalar function, called permittivity and computed from class probabilities as follows:

(1) $$\begin{array}{c} \begin{array}{c} ɛ\left(\vec{r}\;\right)=1+\left({ɛ}_{\max}-1\right)\left(C_{d}\left(\vec{r}\;\right)P_{w}\left(\vec{r}\;\right)+P_{g}\left(\vec{r\;}\right)\right), \end{array} \end{array}$$

where *ɛ*_max⁡_ is the maximum permittivity of the insulating layer (*ɛ*_max⁡_ should be ≫1 in order to emphasize the inhomogeneity of the dielectric layer; *ɛ*_max⁡_ = 100 was used, and *ɛ*_max⁡_ = 1000 was tested with similar results). Thus, permittivity is close to *ɛ*_max⁡_ when WM and/or GM class probabilities are high and is close to 1 when they are low. Note that the WM probability is included above only to ensure a proper transition of the field near the WM/GM interface, where some border voxels can be classified with low GM but high WM probability, for example, because a smoothed interface can slightly deviate from the initial WM segmentation. The inclusion of WM probability is therefore limited by the constraint field *C*_*d*_, which is computed by thresholding of the WM chamfer distance transform *D*_cmf_ as *C*_*d*_ = {1  if  *D*_cmf_ < *d*_min⁡_, 0  otherwise}, where the distance threshold *d*_min⁡_ can be set at the lower bound on cortical thickness (*≈*1 mm), just enough to ensure a “high-permittivity” transition via boundary WM voxels to the layer of GM voxels. The potential field is found as a solution of Maxwell's equation for an electric field inside inhomogeneous dielectric medium in the absence of free charges:

(2) $$\begin{array}{c} \nabla \left(ɛ\left(\vec{r}\;\right)\vec{E}\left(\vec{r}\;\right)\right)=\nabla ɛ\nabla \varphi +ɛ\Delta \varphi =0. \end{array}$$

Equation (2) assumes that the dielectric medium has linear and isotropic properties; therefore, *ɛ* is a scalar, not a tensor. Boundary conditions are specified as $\varphi (\vec{r}\in \Omega_{w})=1$ and $\varphi (\vec{r}\in \Gamma (\Omega ))=0$, that is, the potential is set to one in the WM core and is set to zero on the boundary of the image volume. The solution of the PDE $\varphi (\vec{r}\in \Omega \setminus \Omega_{w})$ can be obtained as a steady-state solution (∂*φ*/∂*t* → 0) of a corresponding nonstationary equation:

(3) $$\begin{array}{c} \frac{\partial \varphi }{\partial t}=\nabla ɛ\nabla \varphi +ɛ\Delta \varphi . \end{array}$$

Equation (3) can also be viewed as describing the diffusion in inhomogeneous medium, where $ɛ(\vec{r}\;)$ is a spatially varying but stationary diffusion coefficient and $\varphi (\vec{r},t)$ is the concentration of the diffusing substance. This allows for a different physical interpretation of the model: we seek a steady-state spatial distribution of “particles” diffusing from WM source into the medium with a diffusivity proportional to the GM class probability. Qualitatively, it is expected that “particles” would diffuse more freely in GM; therefore, the lines of the gradient field ∇*φ* would tend to concentrate in the GM compartment. Equation (3) can be discretized and solved iteratively as described by [21], for example, using the Jacobi method [22].

### 2.4. Distance Field and Correspondence Functions

Lines of the potential field *φ* are defined as a family of curves that are at each point tangent to the gradient ∇*φ*. Let $d(\vec{s},\vec{r}\;)$ denote the length of a line segment originating at some point in WM boundary $\vec{s}\in \Gamma (\Omega_{w})$ and ending in point $\vec{r}\in \Omega \setminus \Omega_{w}$. If, for any point $\vec{r}$, there is one and only one streamline passing through it, then $d(\vec{r}\;)$ defines a distance field. It is possible to compute the distance field by integrating trajectories explicitly in a Lagrangian framework. Alternatively, using the method described in Yezzi and Prince [23], the distance field can be found as a solution of a PDE in an Eulerian framework on a fixed grid. We note that ∇*φ*/||∇*φ*|| is the unit tangent field of the potential field *φ*. Then, it can be shown that the distance field *d* must satisfy the following PDE:

(4) $$\begin{array}{c} \frac{\nabla \varphi }{||\nabla \varphi ||}\cdot \nabla d\left(\vec{r}\;\right)=1, \end{array}$$

with the boundary condition $d(\vec{r}\in \Gamma (\Omega_{w}))=0$. Correspondences along streamline trajectories can be computed in a similar way. More specifically, let $\vec{\psi }=\left[\psi_{1}(\vec{r}\;),\psi_{2}(\vec{r}\;),\psi_{3}(\vec{r}\;)\right]$ denote a vector of correspondence functions, which establishes a correspondence between a point in the field domain $\vec{r}\in \Omega \setminus \Omega_{w}$ and a “source” point in the WM boundary $\vec{\psi }\in \Gamma (\Omega_{w})$. These correspondence functions *ψ*_*i*_ can be found as solutions of the following PDE (see [24]):

(5) $$\begin{array}{c} \frac{\nabla \varphi }{||\nabla \varphi ||}\cdot \nabla \psi_{i}\left(\vec{r}\;\right)=0, \end{array}$$

with boundary conditions $\psi_{i}\left(\vec{r}=[x_{1},x_{2},x_{3}]\in \Gamma (\Omega_{w})\right)=x_{i}$, where *i* = 1,2, 3.

The first-order PDEs (4) and (5) can be solved using the numerical implementation described by Yezzi and Prince [23]. In principle, finite spatial discretization may violate the one-to-one correspondence property of the flow by clamping several streamline paths into one point on a grid, so the solutions $d(\vec{r}\;)$ and $\vec{\psi }(\vec{r}\;)$ may experience numerical convergence problems in some grid locations. In practice, we found that such problematic points are very sparse and do not impede numerical convergence in the computational domain at large. These points are usually detected among other “shocks” in the distance field by a skeletonization method (Figure 2, step 5.2), which is described next.

### 2.5. Skeleton of the Sulcal Medial Surface

Inside sulci, streamlines originating from opposite cortical banks collide (due to spatial discretization), which results into shocks in the distance field and into “discontinuities” in the correspondence functions. Shocks or singularities of a distance field *d* are defined as a set of points, where spatial derivatives of the field are not well-behaved, that is, the gradient ∇*d* is not well defined. Such shocks appear as discontinuities or sinks in the field. Note that even though the potential field in our model should be, in theory, free from the sinks (because there are no free charges), they may appear in the distance field due to spatial discretization. Let *S* ⊂ *Ω*∖*Ω*_*w*_, called a skeleton of the distance field, denote a set of points on a grid, where shocks are detected by a numerical procedure. Such numerical procedure can be based on finite difference approximations to ∇*d*, as described by Han et al. [1]. The observation is that a centered finite difference numerical scheme will produce values of ||∇*d*|| that are significantly lower than 1 on the shock points and are close to unity elsewhere. Then, the skeleton can be detected as $S=\left\{\vec{r}|(\vec{r}\in \Omega \setminus \Omega_{w})\wedge (d(\vec{r}\;)>d_{\min})\wedge (||\nabla d(\vec{r}\;)||<T)\right\}$, where *d*_min⁡_ is a minimum distance parameter set at the lower bound on cortical thickness and *T* is a specified threshold value (*T* < 1; values *d*_min⁡_ = 1 mm and *T* = 0.8 can be used, similarly to ACE in [1]). We found that the skeleton can be robustly detected by a novel algorithm based on the analysis of the correspondence function [14]. Recall that $\vec{\psi }({\vec{r}}_{0})$ is a vector with coordinates of the streamline's source point at WM boundary. A streamline collision can be detected if, in the neighborhood of ${\vec{r}}_{0}$, there are correspondences to source points that are “distant” between themselves. More formally, the skeleton can be determined as $S=\left\{\vec{r}|(\vec{r}\in \Omega \setminus \Omega_{w})\wedge \max_{i}||\vec{\psi }(\vec{r})-\vec{\psi }({\vec{r}}_{i})||>D_{\min}\right\}$, where ${\vec{r}}_{i}\in N_{n}(\vec{r}\;)$. We used *D*_min⁡_ = 4 voxels and 6 adjacent points $N_{6}(\vec{r}\;)$ in our computations.

### 2.6. Geometric Deformable Model

**Figure 3:**
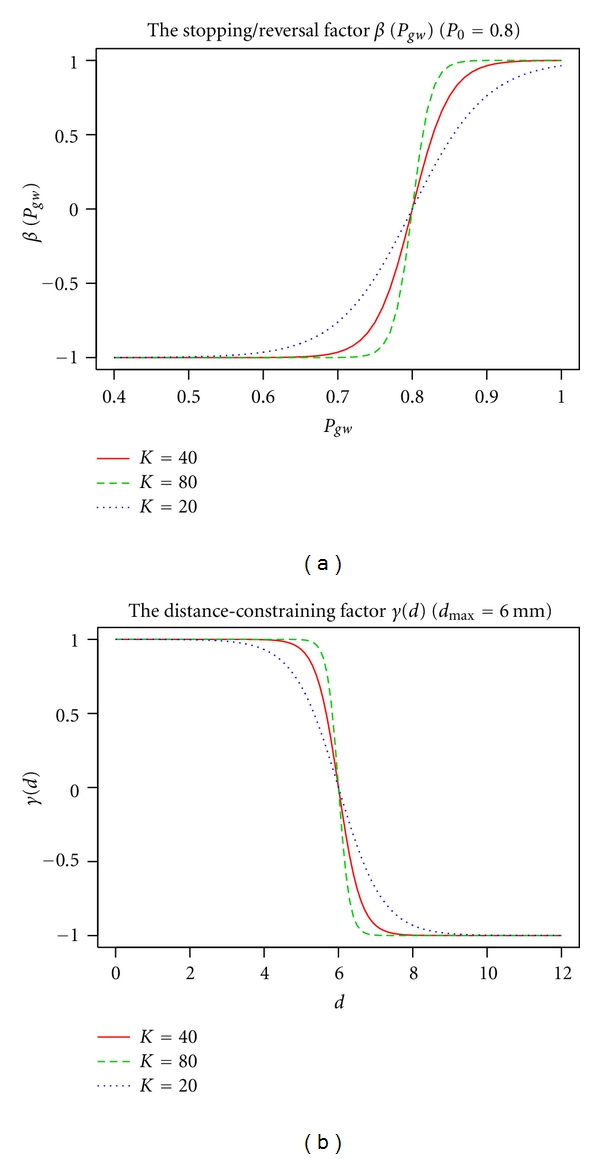
Plots of the stopping/reversal factor *β* (a) and the distance-constraining factor *γ* (b) at different values of the “steepness” constant *K* (solid red line: default *K* = 40; dashed green line: *K* = 80; dotted blue line: *K* = 20).

The geometric deformable model uses an implicit representation of a surface, embedding it into a level set function $\phi (\vec{r},t)$ $\left(\vec{r}\in \Omega \right)$. The evolving interface is represented by the zero-level set $\Phi (t)=\{\vec{r}|\phi (\vec{r},t)=0\}$ (see [16]), and it can be retrieved with subvoxel resolution by an isosurface algorithm (e.g., marching cubes). In our model, evolution of the level set function is described by the following PDE that has an advection and a mean curvature term:

(6) $$\begin{array}{c} \frac{\partial \phi \left(\vec{r},t\right)}{\partial t}+w_{\alpha }\vec{V}\left(\vec{r}\;\right)\cdot \nabla \phi \left(\vec{r},t\right)=w_{\kappa }\kappa \left(\phi \right)||\nabla \phi \left(\vec{r},t\right)||, \end{array}$$

where $\vec{V}$ is the advection velocity vector field, *κ* is the mean curvature, and *w*_*κ*_ are weights of the respective terms (*w*_*α*_, *w*_*κ*_ ≥ 0). The mean curvature of the interface embedded in the level set function is [16]

(7) $$\begin{array}{cc} \kappa & =\nabla \cdot \left(\frac{\nabla \phi }{||\nabla \phi ||}\right)\\ & =(\phi_{x}^{2}\phi_{yy}-2\phi_{x}\phi_{y}\phi_{xy}+\phi_{y}^{2}\phi_{xx}\\ & \;\;+\phi_{x}^{2}\phi_{zz}-2\phi_{x}\phi_{z}\phi_{xz}+\phi_{z}^{2}\phi_{xx}\\ & \;\;+\;\phi_{y}^{2}\phi_{zz}-2\phi_{y}\phi_{z}\phi_{yz}+\phi_{z}^{2}\phi_{yy})/{||\nabla \phi ||}^{3}, \end{array}$$

where the subscripts *x*, *y*, *z* denote partial derivatives. The advection velocity vector field $\vec{V}(\vec{r}\;)$ is derived from the gradient of the potential *φ* or distance field *d*:

(8) $$\vec{V}\left(\vec{r}\;\right)=\{\begin{array}{c} -\beta \left(\vec{r}\;\right)\left(\frac{\nabla \varphi \left(\vec{r}\;\right)}{||\nabla \varphi ||}\right)\\ \text{or}\\ \beta \left(\vec{r}\;\right)\left(\frac{\nabla d\left(\vec{r}\;\right)}{||\nabla d||}\right), \end{array}$$

where $\beta (\vec{r}\;)$ is a stopping/direction-reversal factor computed from the GM/WM class probabilities. For example, this factor can have a form of a logistic function:

(9) $$\begin{array}{c} \beta \left(\vec{r}\;\right)=\frac{2}{1+\exp\left(-K\left[P_{gw}\left(\vec{r}\;\right)-P_{0}\right]\right)}-1, \end{array}$$

where *K* is the constant controlling the steepness of the slope of the sigmoid curve and *P*_0_ is the GM class probability threshold value that determines the “set-point” of the deformable model.  Figure 3 illustrates how the factor *β* depends on GM and WM probability *P*_*gw*_. In our experiments, a moderately steep sigmoid curve with *K* = 40 and the threshold *P*_0_ = 0.8 were used. For spatial regularization, the combined GM and WM class probability $P_{gw}({\vec{r}}_{0})$ can be calculated as a weighted sum over the (closed) neighborhood of the point ${\vec{r}}_{0}$:

(10) $$\begin{array}{c} P_{gw}\left({\vec{r}}_{0}\right)=\underset{{\vec{r}}_{i}\in \left\{{\vec{r}}_{0},\;N_{n}\left(\vec{r}\right)\right\},\;{\vec{r}}_{i}\notin S}{\sum }w_{i}\left(P_{g}\left({\vec{r}}_{i}\right)+P_{w}\left({\vec{r}}_{i}\right)\right), \end{array}$$

where *w*_*i*_ are the neighborhood weights (e.g., *w*_*i*_ = 0.5/*n*, where *n* = 18 or 26, and for the central point *w*_0_ = 0.5), and the skeleton of the sulcal medial surfaces *S* is used for masking of class probability values in separating barriers. As an option, the stopping factor *β* in (8) can be modified to include the distance-constraining factor:

(11) $$\begin{array}{c} \beta_{1}=\left|\beta \left(\vec{r}\right)\right|\left|\gamma \left(\vec{r}\right)\right|\mathrm{sgn}\left(\beta ,\gamma \right), \end{array}$$

where the sign function is an “OR” combination of two signs:

(12) $$\begin{array}{c} \mathrm{sgn}\left(a,b\right)=\{\begin{array}{cc} -1, & \text{if}\;a<0\;\text{or}\;b<0,\\ 1, & \text{otherwise}, \end{array} \end{array}$$

and the distance-constraining factor *γ* can also have a form of a logistic function:

(13) $$\begin{array}{c} \gamma \left(\vec{r}\;\right)=\frac{2}{1+\exp\left(-K\left[1/2-\min\left(d\left(\vec{r}\right),2d_{\max}\right)/2d_{\max}\right]\right)}-1. \end{array}$$

In (13), *d*_max⁡_ is a parameter constraining the maximum distance of advection along the streamlines of the gradient field (i.e., a proximity constraint that limits the thickness of the reconstructed cortical layer). We used *d*_max⁡_ = 6 mm (see Figure 3) in the reported cortical reconstructions, that is, the maximum distance constraint was set above the anatomically plausible upper bound on cortical thickness and therefore was affecting only the artefactual or noncortical gray matter areas.

Our numerical implementation for solving the level set (6) is based on the narrow-band algorithm [12, 16, 25]. The initial level set function is computed as a signed-distance function (SDF) of the initial interface in the corrected WM image using the fast marching method (FMM, [16, 26]). By standard convention, “inside” points are represented by negative values of the SDF. During the evolution, the level set function $\phi (\vec{r},t)$ is maintained close to the SDF by periodic reinitialization with the FMM. The advection term in (6) is discretized based on the upwind differencing scheme (for details, see [16]), and the curvature term is discretized along the lines of (7) using the central differencing scheme [22]. A pseudocode outlining the narrow-band algorithm is described elsewhere (e.g., in [12, 25]). In Algorithm 1 pseudocode we focus on the core part that deals with the time-step update of the level set function. The update algorithm is novel in the way it uses the skeleton of the sulcal medial surface to create barriers for the evolving interface. In addition, the algorithm has a built-in rule preserving the digital topology of the deformed model [1, 12] that is based on the concept of simple points [27] (function IsSimple() in Algorithm 1, see details in [13]), which guarantees that the deformed surface retains the same topology as the initial WM/GM surface.

As already mentioned, the inner cortical surface is reconstructed by a few iterations of the model with the curvature term only (*w*_*α*_ = 0, *w*_*κ*_ = 1) (Figure 2, step 5.0). In step 5.3 of Figure 2, the outer cortical surface is first reconstructed by a model using the advection term only (*w*_*α*_ = 1, *w*_*κ*_ = 0) until convergence (i.e., until the relative amount of change in the SDF per iteration becomes small, for example, lower than 10^−4^) or for a specified number of time steps and then smoothed by a few iterations with the curvature term, similarly to the inner surface.

## 3. Experiments and Results

Our algorithm was implemented in C++ in the Linux environment and ran on a PC with 2.5 GHz AMD-64 CPU and 4 GB RAM, unless otherwise noted. The algorithm's performance was evaluated on simulated test cases with a simplified geometry of a sulcus, on simulated MRI datasets, on standard resolution T1-weighted MR images of human brains, and on high-resolution (sub-mm) MR images of extracted brain hemispheres.

### 3.1. Simulated Data

**Figure 4:**
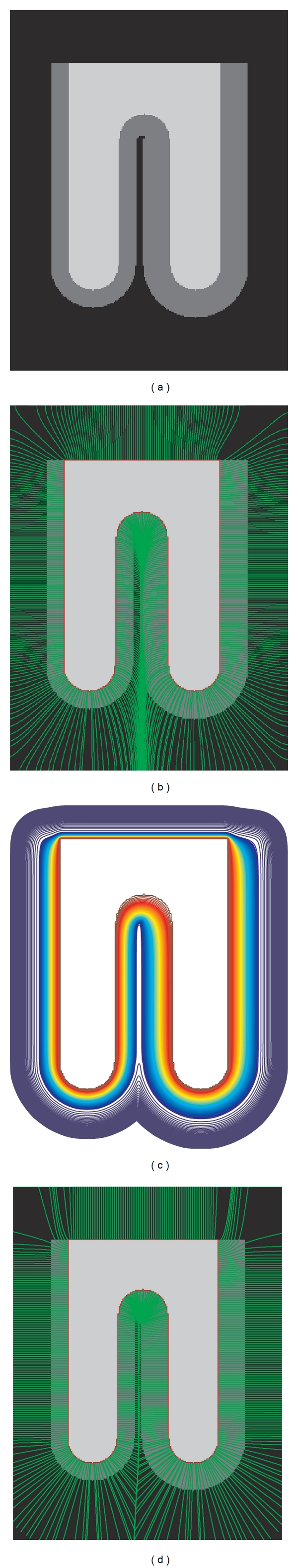
Cross-sections of simulated test images. (a) The input image; (b) field lines in the uniform permittivity model (Laplace equation). Bottom row: isocontours (c) and field lines (d) in the DELFMAP model with the dielectric layer (dark gray in the input image).

The first test case is intended to illustrate the effect of the inhomogeneous dielectric model used in DELFMAP and shows the difference between the field produced with a nonuniform permittivity and the field computed with the uniform permittivity (*ɛ* = 1, the Laplacian field). Test images simulate a simplified 3D geometry of a sulcal fold and contain two WM stalks separated by the sulcal space (with a curvature radius of 10 mm); the WM is covered by a layer of GM having unequal thickness at the opposing banks and a smoothly varying thickness at the fundus (Figure 4(a)).  Figure 4 shows the lines of the Laplacian field (Figure 4(b)) and the lines (Figure 4(d)) and isocontours (Figure 4(c)) of the field in the DELFMAP model. It can be seen that the “ridge” (where the field lines concentrate and the isocontours converge) of the DELFMAP field is close to the sulcal center line, whereas the “ridge” of the Laplacian field is at the geometric center.

**Figure 5:**
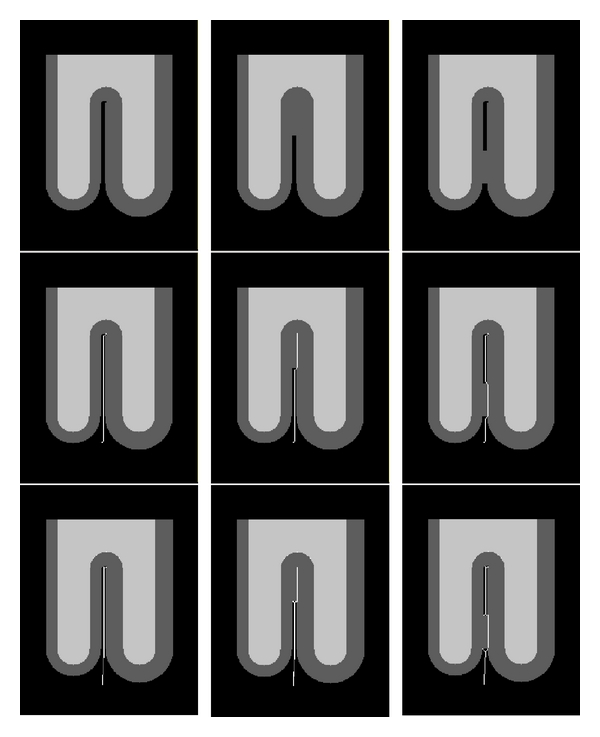
Cross-sections of simulated test images (left: fully resolved sulcus; middle: unresolved fundus; right: bridged sulcus). The white line shows the location of the identified sulcal medial surface skeleton. Comparison of DELFMAP (middle row) versus ACE (bottom row) shows that skeletons produced by DELFMAP have a more regular structure compared to ACE skeletons, which can have small extraneous branches and discontinuities. In the bottom row (ACE), small spurious components are visible at the fundus very close to WM, which in ACE method have to be suppressed by thresholding the distance from WM.

The second test case demonstrates how the model resolves the barrier separating the two opposing cortical banks inside a sulcus. Test images simulate a fully resolved sulcus (with two banks fully separated by background), a sulcus with an unresolved fundus, and a sulcus with two banks bridged by unresolved voxels (the top row in Figure 5: left, middle, and right, resp.). The middle row in Figure 5 shows the cross-section of the sulcal medial surface (white lines) that was identified by the DELFMAP method. It can be seen that the method is capable of reconstructing the boundary surface separating the two cortical banks and finds a geometrically plausible solution in incompletely resolved cases. Side-by-side comparison of the results of our method and those of ACE (the bottom row in Figure 5) shows that skeletons produced by DELFMAP have a more regular structure, whereas ACE skeletons can have small extraneous branches and discontinuities. Our method does not produce spurious detections very close to WM and thus does not require a minimum distance cut-off parameter, which is needed in ACE. In addition, our method is more robust with respect to noise (see [14]): skeletons produced by DELFMAP show very little degradation even at the highest noise level, while ACE skeletons are significantly affected by strong levels of noise.

Cortical reconstruction results for simulated brain phantom MR images [28] showed good reproducibility across various levels of simulated Gaussian-distributed noise and intensity inhomogeneity (see [13, 14]).

### 3.2. High-Resolution MR Images

**Figure 6:**
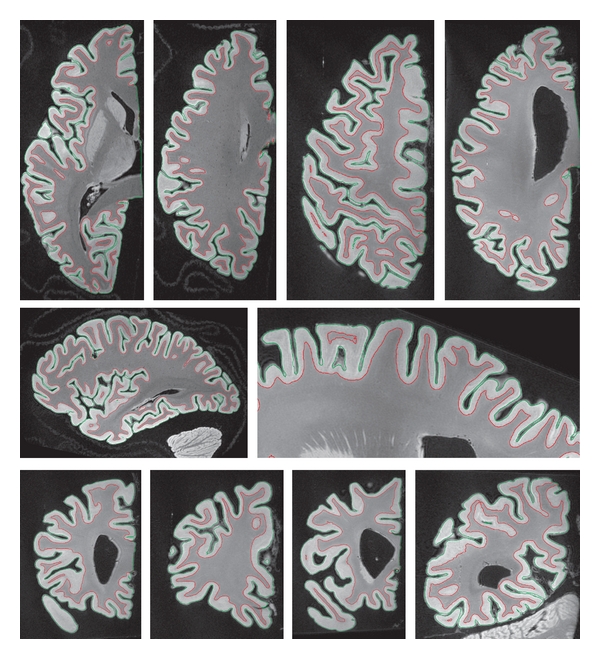
Isocontours of the zero level sets of reconstructed cortical surfaces overlaid on cross-sections of high-resolution MR images (red: the inner surface; green: the outer surface; top, middle, and bottom rows: examples of axial, sagittal, and coronal sections (not to scale), resp.).

Our method's performance is illustrated by results for high-resolution exvivo images, where, contrary to FreeSurfer, our method does not need to conform images to standard 1 mm isotropic voxel size. The algorithm was evaluated on three high-resolution (0.25–0.35 mm isotropic voxel size) images of explanted brain left hemispheres. DELFMAP reconstruction at 0.35 mm resolution took 67 min on a PC with 2.5 GHz AMD-64 CPU and 4 GB RAM. We tried to process the same 0.35 mm data with the recently released CRUISE plugin for MIPAV [29] on a cluster node with four Opteron 285 2.6 GHz cores and 32 GB RAM. Reconstruction of the inner surface took 28 min using 4.9 GB RAM, computation of GGVF took 32 min using 3.5 GB RAM, while reconstruction of the central and pial surfaces took 49 and 52 min using 5.3 and 5.1 GB, respectively, but did not produce adequate results with the default settings. DELFMAP computations at 0.25 mm resolution required 4.7 GB RAM and were successfully completed after 3 h 20 min. Examples of the reconstructed cortical surfaces overlaid on orthogonal cross-sections of a high-resolution MR image are shown in Figure 6.

**Figure 7:**
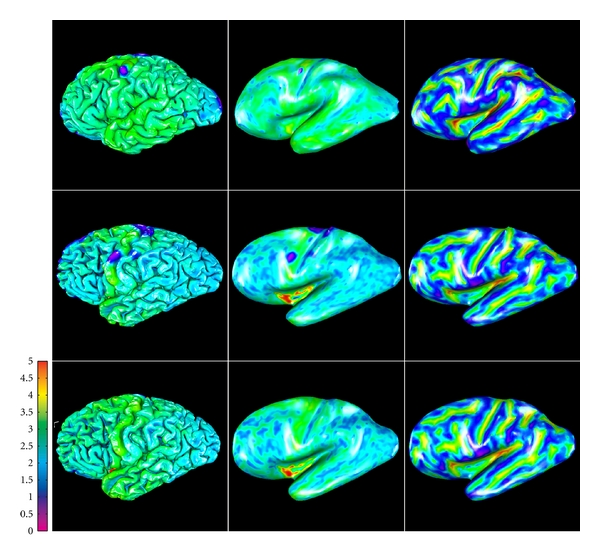
Lateral view of pial surfaces from three high-resolution datasets (left column: thickness maps; middle column: inflated thickness maps; right column: inflated convexity maps).

Lateral views of pial surfaces of three brain samples (3D rendering of thickness maps) are shown in Figure 7, left column. Measured thickness values (mean 2.2 mm; stdev 0.7 mm) are in good agreement with the literature. Inflated maps (Figure 7 middle and right column) are intended for better visualization of the surface inside sulci; they were produced with 20 iterations of Laplacian smoothing of the mesh. Maps in the right column are color-coded with convexity values that were computed as vertex travel distances during smoothing/inflation, similarly to FreeSurfer [4]. On convexity maps, gyral crowns appear in blue color and sulcal fundi appear in yellow-orange. Thickness and convexity maps demonstrate noticeable correlation (Pearson's correlation coefficient computed over the entire surface mesh is 0.24, 0.22, and 0.28 for the three brain samples shown, that is, significantly different from zero at the 0.05 level), which is in good agreement with the known anatomical fact that cerebral cortex is generally thicker on gyral crowns and thinner in sulcal depths.

### 3.3. Cross-Validation with FreeSurfer: Test-Retest Precision

Our method was validated by cross-comparison of cortical reconstruction results with those obtained using FreeSurfer. Standard resolution images for 20 right-handed healthy young subjects (age 19–34, average 23; 8 males/12 females) were selected from the cross-sectional OASIS database [15]. For each subject, data are available from two scan sessions (test and retest) separated by a short delay (1–89, average 21 days), with four T1-weighted standard resolution images acquired per session. This relatively short delay between two consecutive scan sessions makes data sets suitable for the assessment of test-retest reproducibility (i.e., precision) of the analysis by comparing measurements between scan sessions.

**Figure 8:**
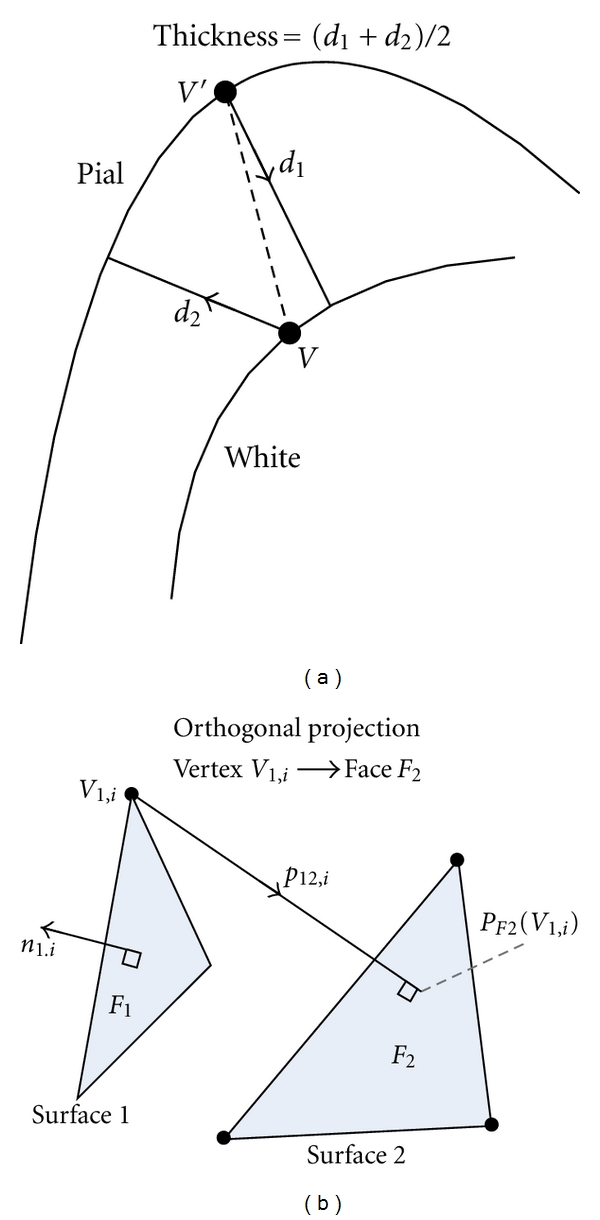
Illustration of two different approaches of defining a distance between two surface meshes. (a) The thickness measure defined in FreeSurfer (2D schematic drawing). (b) The (signed) distance measure defined by closest orthogonal projection.

First, we analyzed data sets using the default automated pipeline in FreeSurfer and obtained 40 cortical reconstructions (two per subject), each including a pial and a white surface mesh. Next, we exported images of extracted brains (without any intensity normalization/correction) and corrected WM segmentations from FreeSurfer, ran our tissue classification algorithm on images of extracted brains, and used these results in the DELFMAP toolchain to obtain another set of 40 cortical reconstructions. For a subvoxel resolution of a digital skeleton, solutions of PDE in (3)–(6) were computed on a grid with half-voxel spacing. Implicit level set surfaces were tessellated using connectivity-consistent marching cubes algorithm [12], and triangular meshes were simplified down to 300,000 faces by a topology-preserving variant of the mesh simplification method [30]. DELFMAP processing took approximately 30 min per brain hemisphere (at half-voxel 0.5 mm res. grid) and was twice faster than FreeSurfer's deformable model step (mris_make_surfaces program, took *≈*70 min at 1 mm res.). FreeSurfer computes cortical thickness at each vertex as the average of the closest-point distance (Figure 8(a)) measured between the surfaces both ways using linked vertices [6]. Since vertices on pial and white surfaces are not linked in DELFMAP, which is not based on a deformable mesh model, for the cross-method comparison, we recomputed cortical thickness using an orthogonal projection distance measure [31] (Figure 8(b) and the Appendix) that is robust and universally applicable to results from both methods. We verified that the two cortical thickness measures were in close agreement on all 40 reconstructions obtained with FreeSurfer.

The geometric precision or test-retest reproducibility of cortical reconstruction was evaluated independently for FreeSurfer and for DELFMAP as follows. For each subject, test and retest MR images (averages of 4 aligned scans from the first and the second session, resp.) were rigidly registered to each other using FSL FLIRT [32]. The obtained rigid transformation was applied to the first set of surface meshes, aligning the test surfaces to the retest ones. Next, signed and absolute distances (the Appendix, (A.1) and (A.2)) were measured between aligned test and retest white/pial surface meshes, and surface-wise mean and standard deviation were computed, as well as the group-wise statistics. In addition, we evaluated the test-retest precision of cortical thickness measured with FreeSurfer and with our method using the standard methodology described in the cortical thickness reproducibility study in Han et al. [33], which consists of the following four steps: (1) rigid registration of two repeated scans of each subject; (2) computation of a thickness difference map for each subject (on the first surface, using point-correspondences established according to closest Euclidean distance in registered space); (3) resampling the thickness difference map to a common template (e.g., any subject surface or the FreeSurfer's average template); (4) computing the group-wise mean and standard deviation of the differences at every vertex of the template mesh. Resampling to a common template relies on FreeSurfer's intersubject registration by nonlinear surface morphing [34].

Results of both methods, the absolute distance measure AD_mean_ and AD_stdev_ between test and retest cortical surfaces (the Appendix, (A.3)), per subject hemisphere, were compared statistically using a Wilcoxon signed rank test, and results are reported as *P* values. For FreeSurfer WM surfaces, reproducibility is characterized by mean absolute error 0.19(Δ0.06) mm (where the Δ value in parentheses indicates a statistical spread for the group, equal to two stdev). For DELFMAP WM surfaces, mean absolute error is 0.24(Δ0.06) mm (*P* = 9.5 × 10^−5^). For DELFMAP pial surfaces, reproducibility is characterized by a mean absolute error 0.24/0.25(Δ0.04) mm (L/R) that is similar in FreeSurfer (L: *P* = 0.37, R: *P* = 0.16, see details in Table 1). The standard deviation of the absolute distance AD_stdev_ is much lower in DELFMAP than in FreeSurfer (L: *P* = 8.2 × 10^−5^, R: *P* = 3.2 × 10^−4^) which can be interpreted as a “tighter” reconstruction of pial surfaces in DELFMAP. Table 1 summarizes the statistics of the test-retest analysis. The mean absolute difference of the cortical thickness is similar in both methods (L: *P* = 0.10, R: *P* = 0.28), but the corresponding standard deviation is again much smaller in DELFMAP than in FreeSurfer (L: *P* = 1.9 × 10^−6^, R: *P* = 1.0 × 10^−4^). To summarize, test-retest precision of cortical thickness measurement is similar in DELFMAP and FreeSurfer in terms of the mean error, which is close to a quarter of the voxel size, but is “tighter” in DELFMAP in terms of surface-wise variance in absolute differences.

### 3.4. Cross-Validation with FreeSurfer: Intermethod Accuracy

The geometric accuracy of our method was evaluated by cross-comparison with FreeSurfer as follows. For each cortical reconstruction (two per subject), white (W) and pial (G) surfaces (Wf, Gf) were exported from FreeSurfer and a cortical thickness map *𝒜*_GfWf_ (A.2) was computed on pial surface. Next, maps of intermethod geometric differences (*𝒟*_WfWd_, *𝒟*_WdWf_, *𝒟*_GfGd_, *𝒟*_GdGf_) were computed as signed distances (A.1) between white or pial surfaces reconstructed with FreeSurfer and DELFMAP (Wd, Gd). On these geometric-difference maps (40 sets, four maps per set), surface-wise statistics D_mean_, D_stdev_, AD_mean_, and AD_stdev_ (A.3) were computed. In addition, maps of intermethod thickness differences were built using the cortical thickness reproducibility analysis steps 2–4 [33] as described in the previous section, except for using two pial surfaces from both methods in step 2 (we emphasize that for both FreeSurfer and DELFMAP, the compared thickness maps were measured by the same method, that is, as *𝒜*_GW_). The 40 individual maps were resampled to a common template and averaged into group-wise maps of mean difference and standard deviation. The group-wise maps of intermethod cortical thickness measurement differences allow to assess and visualize any regional patterns of agreement/disagreement between the two methods. The intermethod geometric accuracy analysis statistics is summarized in Table 2 (averaged over 40 image sets, two per subject). It can be seen from the mean signed distance SD_mean_ that, on average, DELFMAP has a very small outward bias in pial surfaces (−0.08/−0.07(Δ0.08) mm, L/R; negative sign means FreeSurfer' surface is “inside” w.r.t. DELFMAP' surface). The intermethod accuracy can be characterized by the mean absolute distance AD_mean_ (0.40/0.42(Δ0.04) mm, L/R), which is less than a half of the voxel size. The share of pial surface vertices where the AD was larger than 1 mm is less than 10%; less than 1% of pial vertices had an AD larger than 2 mm.

**Figure 9:**
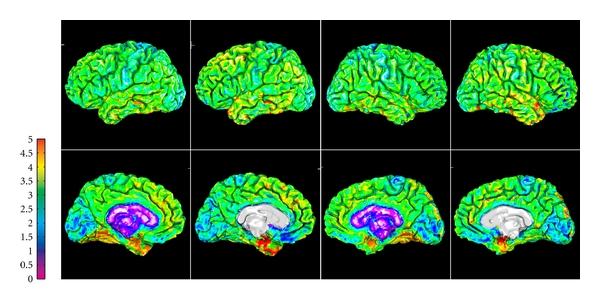
Example of side-by-side comparison of DELFMAP (column 1 and 3) and FreeSurfer (column 2 and 4) thickness maps (OAS1_202_1, on pial surfaces, left/right hemisphere in the left/right two columns, resp.; 1st row: lateral surface; 2nd row: medial surface; colorbar range 0–5 mm).

**Figure 10:**
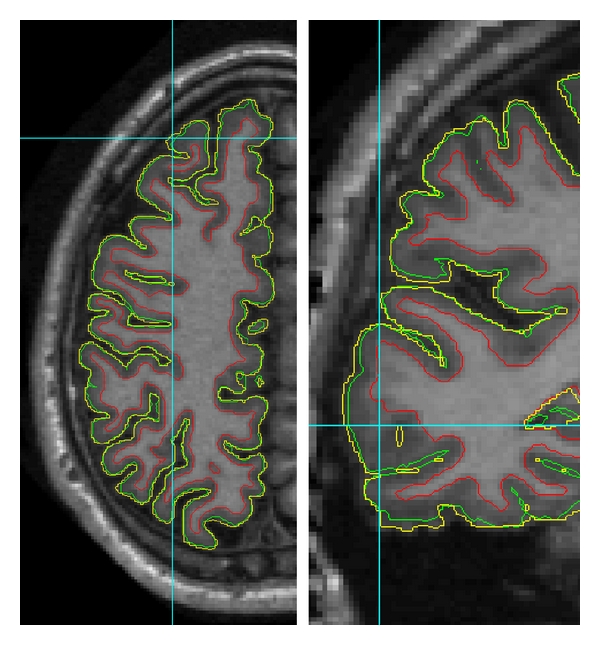
Contours of reconstructed cortical surfaces overlaid on the axial (left) and coronal (right) slice (red: inner surface; green: DELFMAP pial surface; yellow: FreeSurfer pial surface; note that the yellow contour appears jagged because it is displayed from FreeSurfer's volumetric signed distance function sampled at 1 mm grid, whereas red and green contours are from level set functions sampled at a finer resolution; left and right images are not to scale). On the left image at the cross-line cursor position (superior frontal region), the yellow contour of FreeSurfer's pial reconstruction oversegments into meningeal space, and a similar trend is noticeable next to cursor on the right image (temporal region).

The intermethod accuracy analysis of cortical thickness measurements, summarized in Table 3, is in good agreement with the above observations. On average, there is a small bias towards thicker values in DELFMAP (mean signed difference: 0.12/0.11(Δ0.16) mm, L/R; positive sign here means that DELFMAP-measured thickness is larger w.r.t. FreeSurfer). The intermethod accuracy, characterized by the mean absolute difference (0.35/0.34(Δ0.06) mm, L/R), is less than a half of the voxel size. The share of pial surface vertices where the absolute difference between thickness measurements was larger than 1 mm is less than 6%, and less than 1% of pial vertices had an absolute difference larger than 2 mm. An example comparing DELFMAP and FreeSurfer pial surface reconstructions side-by-side, for one subject, is shown in Figure 9 (colored with cortical thickness; see colorbar for color map and range of values). Overall, a good correspondence is visible, but some patterns of thickness difference are noticeable: (1) for FreeSurfer, thickness is larger (indicated as yellow) in the superior region of the frontal lobe and in some temporal regions (lateral view); (2) for DELFMAP, thickness is larger (indicated as orange) in the inferior occipitotemporal region (medial view, where the cerebellum is found); (3) for FreeSurfer, thickness is smaller (indicated as blue) in the medial orbitofrontal cortex (mOFC) region (medial view). These differences can be attributed and traced to the following segmentation trends in either of the two methods: (1) oversegmentation, by FreeSurfer, into meningeal space in superior frontal region and in temporal region (see Figure 10); (2) oversegmentation, by DELFMAP, into cerebellar gray matter in the inferior occipitotemporal region; (3) too conservative segmentation, by FreeSurfer, in the mOFC region (too thin, less than 1.5 mm).

**Figure 11:**
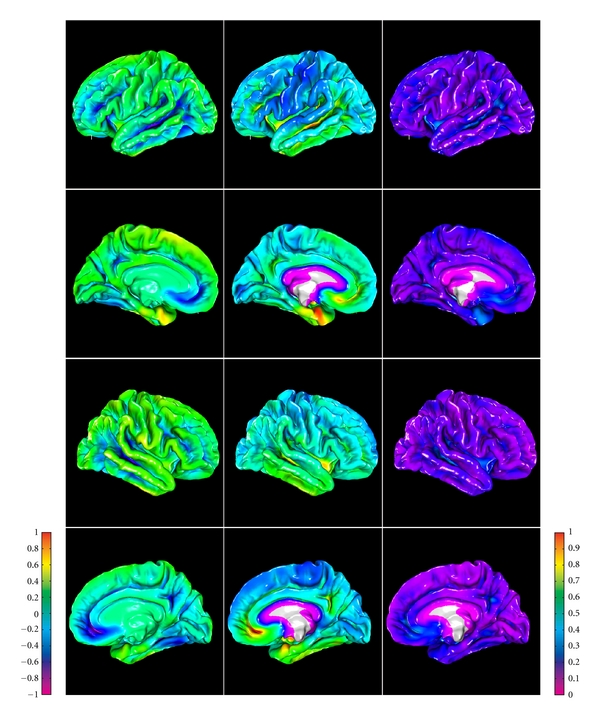
Group-average maps of intermethod (DELFMAP-FreeSurfer) geometric differences in pial surface reconstructions, resampled to FreeSurfer's average template (left column: signed distance mean, colorbar range ±1 mm, negative/positive values mean FreeSurfer' surface is inside/outside of DELFMAP' surface, resp.; middle column: absolute distance mean, colorbar range 0-1 mm; right column: absolute distance stdev., colorbar range 0-1 mm; rows 1–4: lateral/medial surface of left/right hemisphere, resp.).

**Figure 12:**
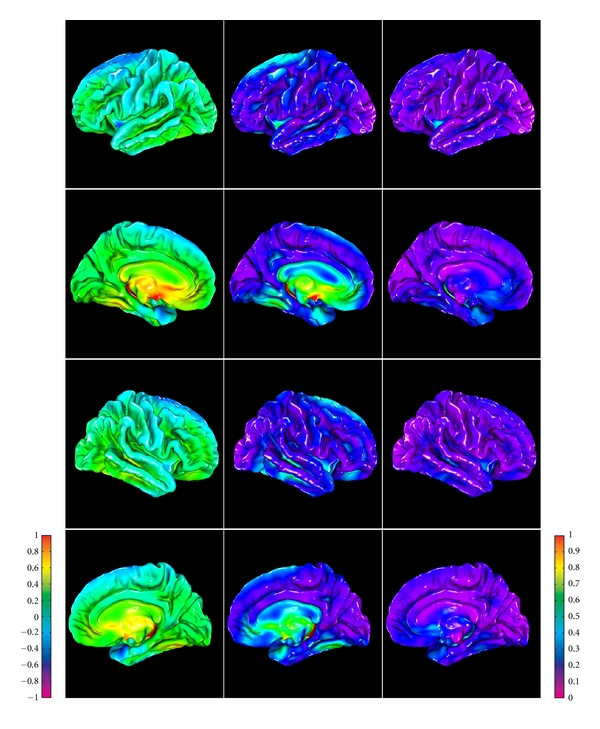
Group-average maps of intermethod (DELFMAP-Freesurfer) cortical thickness differences, resampled to FreeSurfer's average template (left column: signed difference mean, colorbar range ±1 mm, negative/positive values mean thickness measured with DELFMAP is smaller/larger than measured with FreeSurfer, resp.; middle column: absolute difference mean, colorbar range 0-1 mm; right column: absolute difference stdev., colorbar range 0-1 mm; rows 1–4: lateral/medial surface of left/right hemisphere, resp.).

Regional patterns of intermethod geometric differences in pial cortical reconstructions are visible on group-average maps of geometric (Figure 11) and cortical thickness differences (Figure 12), where the above outlined three trends are also noticeable.

## 4. Discussion

We presented a novel PDE-based approach for reconstructing the cerebral cortex from MR images. We developed an accurate and scalable method that works on MR images with a high spatial resolution. Because high-resolution MRI begins to attract considerable attention in brain imaging research, a method that readily scales with imaging resolution is highly valuable. This scalability is achieved by using an implicit deformable surface model in a fast marching framework guided by a novel, computationally efficient model using potential field mapping. Our method requires much lower computational resources and has a much faster computation times than conventional methods. These demonstrated advantages come not only from an efficient practical implementation, but also from the design of our algorithms. For instance, other existing approaches that are based on deformable mesh models incur a significant computational cost associated with the mesh self-intersection (e.g., FreeSurfer) or mesh self-proximity (CLASP) term, which does not scale linearly with increasing mesh resolution. Although the computational cost of the straightforward mesh self-proximity term [2], which is quadratic *O*(*N* ^2^/2) on the number of faces *N*, is significantly reduced in a mesh self-intersection prevention algorithm utilizing a spatial cache [3], it nevertheless remains supralinear. Similarly, the cost of another known efficient algorithm for mesh self-intersection detection, which is based on intersection of bounding boxes, is *O*(*N*log⁡_2_^3^ *N*) [35]. In contrast to this, the computational complexity *O*(*Nk*) of the narrow-band level set algorithm used in our method (and in CRUISE) is linear with respect to the size of the interface *N* (*k* is the width of the narrow band). This difference between a linear and a quadratic or supralinear algorithmic complexity, which can be tolerated when dealing with standard resolution images and meshes, becomes quite large at high resolutions. As to the comparison with the available CRUISE MIPAV software, our method's dramatic gain in speed is most likely due to differences in implementation but, at least in part, can be attributed to a smaller algorithmic cost of our method (e.g., solving one second-order PDE in DELFMAP versus a system of three second-order PDEs in GGVF, and not using an intermediate step of reconstructing a central cortical surface).

Although some algorithmic building blocks of our method were previously known to the medical image processing community (e.g., [1, 21, 23]), the central aspect of our method, that is, the use of the model of the potential field in the inhomogeneous dielectric layer introduced here, is novel and has attractive advantages. The novelty of our method is also in the newly introduced skeletonization algorithm that is based on the analysis of correspondence trajectories and in several novel aspects of the geometric deformable model (e.g., the constraint of the evolution by the medial surfaces, the maximal distance constraint of the advection, and the novel form of the advection stopping/direction-reversal factor *β* and the distance-constraining factor *γ*). We note that most of the design parameters introduced in Section 2 remain fixed, and the method is sensitive only to two settings, which can be easily tuned: the GM probability threshold *P*_0_ (a “set-point”) and the maximal distance *d*_max⁡_ (which has strong influence only if set below the upper bound on cortical thickness).

The results from three high-resolution data sets demonstrate that the method is capable of reconstructing the outer cortical boundary with good geometric precision and accuracy, while guaranteeing the preservation of the initial surface topology. The method's performance is illustrated on synthetic images and on standard resolution MR brain images, where it compares favorably to existing methods in both quality and speed.

The precision and accuracy of our method was assessed by cross-validation in standard resolution datasets with the widely accepted approach implemented in the available FreeSurfer software. Using a database of consecutive examinations in healthy subjects, the precision of both methods was evaluated using pointwise geometric distances of reconstructed surfaces and differences in cortical thickness. Both methods are similar in terms of the mean absolute error in position and mean absolute error in cortical thickness. However, DELFMAP has a much lower variance than FreeSurfer. In a second study, we evaluated the accuracy of our method by quantifying the intermethod reproducibility of reconstructed cortical surfaces, measured by pointwise geometric distances and differences in cortical thickness measurement between the two methods. Results demonstrate that the accuracy of our method, using FreeSurfer as a reference, is better than half of a mm in terms of both mean absolute error in geometric position and mean absolute error in measured cortical thickness. Group-average analysis of the spatial distribution of geometric and thickness differences between the two methods reveals some surface regions, where one of the two methods has a tendency to systematically over- or undersegment the cortical ribbon, resulting in patterns of small (subvoxel) but measurable differences. Thus, cross-comparison of the two methods allows detection of existing regional patterns in intermethod differences, benefiting the study of accuracy of both approaches and highlighting some potentially problematic areas for further improvement of both methods.

## Figures and Tables

**Algorithm 1:**
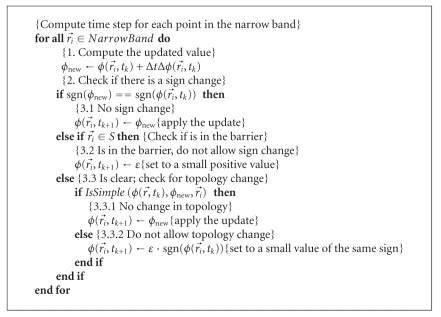
The level set function update algorithm.

**Table 1:** Precision analysis: summary of the group-average statistics for the signed distance (SD) and absolute distance (AD) measure (in mm) between test and retest surfaces (surface: DF—DELFMAP, FS—FreeSurfer; L/R: left/right hemisphere; mean: a group average of a surface-wise mean of the distance; stdev: a group average of a surface-wise stdev of the distance; “>*X* mm (%)”: (group-average) percentage of surface points where AD was greater than *X* mm; values in parentheses indicate the statistical spread within the group, measured by the group-wise stdev).

Surface	L/R	Signed distance		Absolute distance			
		Mean (mm)	stdev (mm)	Mean (mm)	stdev (mm)	>1 mm (%)	>2 mm (%)
DF pial	L	−0.02 (0.03)	0.35 (0.06)	0.24 (0.02)	0.25 (0.04)	1.4 (0.3)	0.2 (0.1)
	R	−0.01 (0.04)	0.37 (0.09)	0.25 (0.02)	0.26 (0.06)	1.5 (0.4)	0.2 (0.2)
FS pial	L	−0.02 (0.04)	0.37 (0.10)	0.24 (0.02)	0.28 (0.06)	1.9 (0.4)	0.3 (0.2)
	R	−0.03 (0.04)	0.39 (0.13)	0.24 (0.03)	0.29 (0.08)	2.0 (0.4)	0.3 (0.2)
DF white	L	−0.01 (0.07)	0.34 (0.08)	0.24 (0.02)	0.24 (0.05)	1.0 (0.3)	0.2 (0.1)
	R	+0.02 (0.06)	0.35 (0.11)	0.24 (0.03)	0.24 (0.07)	1.0 (0.3)	0.2 (0.2)
FS white	L	+0.02 (0.02)	0.31 (0.10)	0.19 (0.02)	0.23 (0.07)	1.0 (0.3)	0.2 (0.2)
	R	+0.01 (0.02)	0.31 (0.13)	0.19 (0.03)	0.23 (0.08)	0.9 (0.3)	0.2 (0.2)

**Table 2:** Intermethod accuracy analysis: summary of the group-average statistics for distances between DELFMAP- and FreeSurfer-generated surfaces.

Surf.	L/R	Signed distance		Absolute distance			
		Mean (mm)	stdev (mm)	Mean (mm)	stdev (mm)	>1 mm (%)	>2 mm (%)
pial	L	−0.08 (0.04)	0.49 (0.02)	0.40 (0.02)	0.37 (0.02)	6.8 (1.2)	0.6 (0.2)
	R	−0.07 (0.04)	0.53 (0.02)	0.42 (0.02)	0.38 (0.02)	7.4 (1.3)	0.6 (0.2)
white	L	0.00 (0.04)	0.28 (0.01)	0.24 (0.01)	0.17 (0.01)	0.1 (0.1)	0.0 (0.01)
	R	0.00 (0.04)	0.29 (0.01)	0.24 (0.01)	0.18 (0.01)	0.0 (0.0)	0.0 (0.01)

**Table 3:** Intermethod accuracy analysis: summary of the group-average statistics for difference in cortical thickness measurement between DELFMAP and FreeSurfer.

L/R	Signed difference		Absolute difference			
	Mean (mm)	stdev (mm)	Mean (mm)	stdev (mm)	>1 mm (%)	>2 mm (%)
L	0.12 (0.07)	0.47 (0.03)	0.35 (0.03)	0.34 (0.03)	4.4 (1.5)	0.4 (0.1)
R	0.11 (0.08)	0.46 (0.03)	0.34 (0.03)	0.33 (0.03)	4.1 (1.4)	0.3 (0.1)

## Appendix

### Distance Measure between Two Surfaces

The orthogonal projection method [31] was adapted to define a measure of geometric distance between two meshes that was used throughout our validation study. We note that a similar approach was proposed in Tosun et al. [36] for accuracy and precision analysis of cortical surface reconstructions. The signed distance between two triangulated meshes *ℳ*_1_ = {*𝒱*_1_, *ℱ*_1_}_*N*_1__ and *ℳ*_2_ = {*𝒱*_2_, *ℱ*_2_}_*N*_2__ was measured as:

(A.1) $${𝒟}_{12}={\left\{d_{12,i}=\left({\vec{p}}_{12,i}\cdot {\vec{n}}_{1,i}\right)||{\vec{p}}_{12,i}||:{\vec{p}}_{12,i}={\vec{v}}_{1,i}-{𝒫}_{{ℱ}_{2}}\left({\vec{v}}_{1,i}\right)\right\}}_{N_{1}},$$

where ${𝒫}_{{ℱ}_{2}}({\vec{v}}_{1,i})$ is the closest orthogonal projection operator projecting a vertex ${\vec{v}}_{1,i}$ from the first mesh onto one of the triangles *ℱ*_2_ in the second mesh, along the normal to that triangle (Figure 8(b)). The sign of the distance measure is determined by the innerproduct of the projection difference vector ${\vec{p}}_{12,i}$ with the first surface outward normal ${\vec{n}}_{1,i}$ at vertex ${\vec{v}}_{1,i}$; thus, a positive/negative value signifies that the second surface is outside/inside of the first surface, respectively. For the signed distance, mean and standard deviation (stdev) are computed on *d*_12,*i*_ values. We define the absolute distance measure as:

(A.2) $$\begin{array}{c} {𝒜}_{12}={\left\{a_{12,i}=||{\vec{p}}_{12,i}||:\left(a_{12,i}=\left|d_{12,i}\right|\right)\right\}}_{N_{1}}. \end{array}$$

In this notation, the cortical thickness measure of Kruggel and von Cramon [31] is defined as the absolute distance from GM to WM mesh *𝒜*_GW_; this orthogonal projection measure should not be confused with the distance along surface normal [37], which was shown to be less reliable compared to other distance measures [38]. For the absolute distance, two-way mean and standard deviation are computed (see also [36]) as:

(A.3) $$\begin{array}{cc} \text{A}{\text{D}}_{\text{mean}} & =\frac{1}{N_{1}+N_{2}}\left(\overset{N_{1}}{\underset{i=1}{\sum }}a_{12,i}+\overset{N_{2}}{\underset{j=1}{\sum }}a_{21,j}\right),\\ \text{A}{\text{D}}_{\text{stdev}} & ={\left(\frac{1}{N_{1}+N_{2}}\left(\overset{N_{1}}{\underset{i=1}{\sum }}a_{12,i}^{2}+\overset{N_{2}}{\underset{j=1}{\sum }}a_{21,j}^{2}\right)-\text{A}{\text{D}}_{\text{mean}}^{2}\right)}^{1/2}. \end{array}$$
